# Perineural injection therapy as a potential treatment for chronic pain after carpal tunnel surgery: a case report

**DOI:** 10.11604/pamj.2025.50.18.45627

**Published:** 2025-01-08

**Authors:** Devi Ariani Sudibyo, Hanik Badriyah Hidayati, Fidiana, Septika Ekasari, Qisti Ashari

**Affiliations:** 1 Faculty of Medicine, Universitas Airlangga /Dr. Soetomo General Academic Hospital, Jl. Mayjen Prof. Dr. Moestopo No 47 Surabaya 60132, Surabaya, Indonesia

**Keywords:** Carpal tunnel syndrome, perineural injection therapy, chronic postoperative pain, case report

## Abstract

Carpal Tunnel Syndrome (CTS) is a common peripheral neuropathy caused by compression of the median nerve in the wrist. While surgery is often successful, a subset of patients develops Chronic Postoperative Pain (CPOP). This case report discusses a 46-year-old woman who experienced persistent bilateral wrist pain after carpal tunnel surgery, primarily affecting the right hand. Following the recurrence of symptoms after surgery, we administered Perineural Injection Therapy (PIT) using 5% dextrose. The patient experienced significant pain relief, with her Numeric Rating Scale (NRS) score decreasing from 8/10 to 2/10 after three sessions. Additionally, her functional status improved as measured by the Boston Carpal Tunnel Questionnaire (BCTQ). PIT proved to be a non-invasive and effective alternative in managing CPOP when other treatments had failed, demonstrating its potential to improve symptoms and quality of life.

## Introduction

Carpal Tunnel Syndrome (CTS) is caused by the compression of the median nerve in the wrist, leading to sensory disturbances in the first three fingers [1]. When non-surgical therapies are unsuccessful or for moderate-to-severe instances of CTS, surgery could be recommended. Though the majority of patients who have surgery report outstanding results, only 75% of them report better than before, and 8% report advancing symptoms [2,3]. Through lack of improvement in results, complications such as inadequate release of the transverse carpal ligament, scarring, chronic discomfort, or grip weakness may arise [2].

Chronic Postoperative Pain (CPOP) is a recognized complication following major orthopedic surgeries, with an incidence ranging between 8% and 28% for hip replacements and up to 35% for knee replacements. However, studies on minor orthopedic procedures, such as carpal tunnel surgery, are limited. Comparable to the rates seen after more invasive procedures, a research found that one year following carpal tunnel release, 22% of patients reported having CPOP [4].

Recent clinical trials have shown that Perineural Injection Therapy (PIT) with 5% dextrose water (D5W) is a viable long-term treatment for Carpal Tunnel Syndrome (CTS) [5,6]. The therapy works by injecting a 5% dextrose solution into peripheral nerves, targeting neurogenic inflammation with osmolality similar to normal saline. The injections cause less discomfort than sterile water [5,7]. Pharmacological, mechanical, and maybe neuroregenerative effects are among its advantages; they are particularly relevant for peripheral neuropathy brought on by nerve entrapment [5]. One important process is hydrodissection, which reduces compressive damage and improves blood flow by releasing adhesions from the median nerve [8].

## Patient and observation

### Patient information

**De-identified patient information:** a 46-year-old woman presented with chronic bilateral wrist pain, primarily in the right hand.

**Primary concerns and symptoms:** the patient reported electric shock-like pain with numbness, stiffness, and tingling, particularly affecting the first three fingers and half of the fourth on both hands. The pain rated 8/10 on the Numeric Rating Scale (NRS), increased to NRS 10 at night, and significantly affected daily activities and sleep.

**Medical, family, and psychosocial history:** no relevant genetic history was reported. The patient´s condition caused challenges in her daily routines, especially in tasks requiring manual dexterity, such as cooking.

**Relevant past interventions and outcomes:** initially treated with physiotherapy and oral paracetamol, the patient experienced minimal relief. In 2019, she underwent carpal tunnel release surgery on the right hand, with pain initially decreasing to NRS 6/10. However, symptoms recurred one year later, returning to NRS 8 and worsening to NRS 9-10 at night, persisting despite additional conservative therapies.

**Clinical findings:** upon clinical examination, her bilateral motor strength was 5/5 based on the Medical Research Council (MRC) scale, without atrophy. Sensory examination revealed paraesthesia in the first three digits and half of the fourth digit on both hands. Provocative tests including the Tinel sign, Phalen test, Flick sign, and Luthy sign were positive bilaterally. No abnormalities were found in the cranial nerve examination.

**Timeline of the current episode:** onset of bilateral wrist pain, primarily in the right hand, with initial NRS 8/10, worsening to NRS 10 at night four years ago. The pre-surgery period was managed with physiotherapy and paracetamol, with pain reaching NRS 10 and no injection therapy. Difficulty with daily activities and sleep. In 2019: underwent carpal tunnel release surgery on the right hand, resulting in initial pain reduction to NRS 6/10. One year post-surgery (2020): pain recurred with NRS of 8, intensifying at night (NRS 9-10). Persistent impact on sleep and daily activities, especially cooking. Neurological assessment: clinical findings indicated preserved motor strength (5/5), paresthesia, and positive Tinel, Phalen, Flick, and Luthy signs bilaterally. Dextrose PIT initiation (2023): 5% dextrose PIT was administered to the right hand over three sessions at four-week intervals. Injection was performed along the surgical scar and surrounding areas.

### Diagnostic assessment

**Figure 1:**
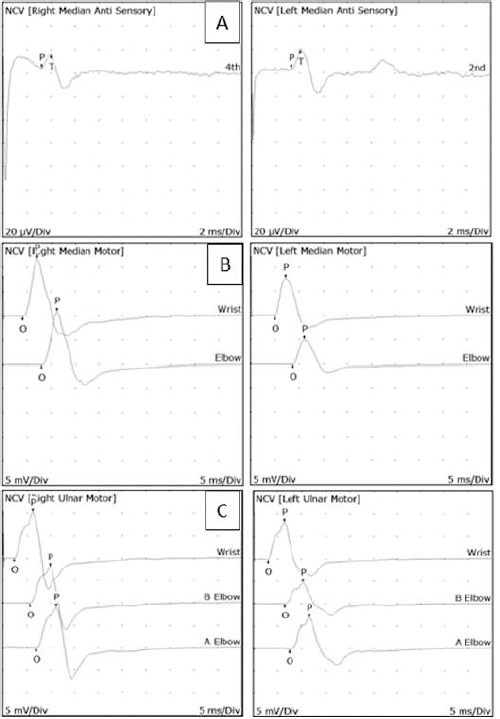
A, B) nerve conduction study (NCS) findings; C) needle EMG shows no spontaneous activity, indicating the absence of acute denervation

**Diagnostic testing:** diagnosis was primarily based on physical examination, Electromyography (EMG), and Nerve Conduction Velocity (NCV) studies, which revealed prolonged distal latency in motor and sensory nerves in both hands, alongside right-sided nerve root irritation at C5, C6, and C7 (Figure 1).

**Diagnostic challenges:** the recurrence of pain post-surgery presented a diagnostic challenge, necessitating additional therapeutic interventions beyond the standard physiotherapy and pharmacologic approaches.

**Diagnosis:** persistent carpal tunnel syndrome with nerve root irritation.

**Prognostic characteristics:** given the recurrence of symptoms post-surgery and minimal response to conservative management, prognosis for pain relief with conservative management alone was poor.

### Therapeutic intervention

**Types of therapeutic intervention:** Perineural Injection Therapy (PIT) with 5% dextrose was selected for pain management. The decision was based on the patient´s resistance to standard therapies and the recurrence of severe symptoms after surgery.

**Administration of therapeutic intervention:** a 5% dextrose injection was administered to the right hand along the surgical scar and adjacent areas in three sessions at four-week intervals. Each injection used 5 ml of dextrose.

**Changes in therapeutic intervention:** due to persistent pain, PIT was combined with continued physiotherapy to optimize recovery.

**Follow-up and outcomes**

**Clinician- and patient-assessed outcomes:** following PIT, the patient´s pain reduced significantly from NRS 8 to 2/10. The Boston Carpal Tunnel Syndrome Questionnaire (BCTQ) scores improved, showing a reduction in symptom severity from 43 (severe) to 29 (moderate) and functional impairment from 18 (severe) to 9 (moderate).

**Follow-up diagnostic and other test results:** no further diagnostic tests were necessary post-intervention. The patient´s functional status and daily activities were assessed through clinical follow-up.

**Intervention adherence and tolerability:** the patient adhered well to the therapy schedule, reporting no adverse effects from the injections. Tolerability was confirmed through follow-up assessments.

**Adverse and unanticipated events:** no adverse or unexpected events occurred during the treatment course.

**Patient perspective:** the patient expressed satisfaction with the treatment, noting significant pain relief and improved functionality, especially compared to the minimal improvement after her initial surgery.

**Informed consent:** it was obtained from the patient for the publication of this case report and accompanying images.

## Discussion

CTS, the most common peripheral entrapment neuropathy, occurs due to excessive compression of the median nerve, leading to ischaemia and eventual nerve damage within the carpal tunnel. This compression primarily causes sensory deficits in the palm, especially affecting the first to third fingers. While motor symptoms may develop as the condition progresses, sensory disturbances are typically more prominent in the early stages [1].

In this case, a 46-year-old woman presented with bilateral wrist pain, predominantly in the right hand, which had persisted for four years despite undergoing carpal tunnel release surgery a year earlier. The pain, rated at NRS 8/10, initially affected the first to half of the fourth fingers on her right hand before spreading to her left hand. Described as electric shock-like, the pain was accompanied by numbness, stiffness, and tingling, worsening at night, and aggravated by activities such as cooking and typing. These symptoms align with findings from a study by Belze *et al*., who reported that 35% of patients continue to experience pain one year post-CTS surgery, with 22% developing CPOP [4].

Post-surgical EMG/NCV diagnostic results showed prolonged distal latency in both motor and sensory nerves in both hands, alongside nerve root irritation at C5, C6, and C7. The patient´s BCTQ scores were 43 (severe) for symptoms and 18 (severe) for functional status, which aligns with Wu YT´s findings that abnormal nerve conduction is an indicator of moderate CTS severity [5]. Long-term studies highlight that while the majority of patients experience lasting relief after surgery, some individuals may develop persistent issues. In such cases, PIT offers a non-invasive alternative with significant improvements in managing CPOP when other interventions have failed. A first retrospective follow-up study's findings, with follow-up durations spanning from 1 to 3 years, revealed that 88.6% of patients saw a successful outcome after a mean of 2.2 injections. Notably, 80% of patients with surgery failure or post-surgery recurrence achieved effective outcomes [3].

In this particular situation, a non-invasive substitute called PIT with 5% dextrose was used. The patient received 5 ml of dextrose via a blind injection technique, chosen for its practicality and the predictable anatomy of the affected area. While ultrasound-guided injections offer greater precision and reduced risks, blind injections remain an effective and accessible option when performed by experienced clinicians. Studies have shown favourable outcomes with blind injections in predictable areas like the carpal tunnel, though ultrasound guidance is often associated with better symptom relief and functional improvement [9].

The mechanism of action of 5% dextrose involves the reduction of neurogenic inflammation by inhibiting capsaicin-sensitive TRPV1 receptors, which are found in peripheral nerves, ligaments, tendons, and joints. TRPV1 upregulation is linked to neuropathic pain, and its downregulation via D5W inhibits the release of pro-nociceptive substances such as substance P and calcitonin gene-related peptide (CGRP). This reduction in neurogenic inflammation likely contributed to the pain relief observed in this case [6,7].

After the first injection, the patient reported significant pain reduction, with the NRS dropping from 8 to 3. Even to the third session, the injections which usually targeted the median nerve and the ulnar neurovascular bundle, deep in the palmaris longus (PL) tendon were repeated every four weeks [10]. By the third injection, the pain had further reduced to an NRS of 2, and the patient´s BCTQ scores improved to 29 for symptoms and 9 for functional status (moderate). She regained the ability to perform daily activities such as holding keys, writing, and using a cell phone, though some difficulty persisted with circular movements like opening jars. The efficacy of multiple dextrose injections in managing CPOP and improving functional status [3].

## Conclusion

Carpal Tunnel Syndrome (CTS) is a common peripheral entrapment neuropathy caused by excessive compression of the median nerve, leading to nerve damage within the carpal tunnel. While most patients experience relief after surgery, a minority may continue to suffer from pain, which can evolve into Chronic Postoperative Pain (CPOP). In this case report, Perineural Injection Therapy (PIT) with 5% dextrose has proven to be an effective non-invasive alternative for addressing persistent pain following surgery. Although ultrasound-guided injections offer greater precision, blind injections remain a practical and effective option. These findings support the use of PIT as a valuable adjunct in managing postoperative pain, providing significant benefits in symptom relief and functional recovery.
